# *Anacardium microcarpum* Promotes Neuroprotection Dependently of AKT and ERK Phosphorylation but Does Not Prevent Mitochondrial Damage by 6-OHDA

**DOI:** 10.1155/2018/2131895

**Published:** 2018-10-29

**Authors:** Illana Kemmerich Martins, Nélson Rodrigues de Carvalho, Giulianna Echeverria Macedo, Nathane Rosa Rodrigues, Cynthia Camila Ziech, Lúcia Vinadé, Valter Menezes Barbosa Filho, Irwin Alencar Menezes, Jeferson Franco, Thaís Posser

**Affiliations:** ^1^Oxidative Stress and Cell Signaling Research Group, Centro Interdisciplinar de Pesquisa em Biotecnologia, Universidade Federal do Pampa, Campus São Gabriel, 97300-000 São Gabriel, RS, Brazil; ^2^Instituto Federal Farroupilha, Campus Santo Ângelo, 98806-700 Santo Ângelo, RS, Brazil; ^3^Departamento de Química, Programa de Pós-Graduação em Bioquímica Toxicológica, Universidade Federal de Santa Maria, 97105-900 Santa Maria, RS, Brazil; ^4^Departamento de Química Biológica, Universidade Regional do Cariri, 63100-000 Crato, CE, Brazil

## Abstract

Parkinson's disease is a degenerative and progressive illness characterized by the degeneration of dopaminergic neurons. 6-hydroxydopamine (6-OHDA) is a widespread model for induction of molecular and behavioral alterations similar to Parkinson and has contributed for testing of compounds with neuroprotective potential. The Brazilian plant *Anacardium microcarpum* is used in folk medicine for treatment of several illnesses; however, the knowledge about toxicology and biological effects for this plant is very rare. The neuroprotective effect from hydroalcoholic extract and methanolic and acetate fraction of *A. microcarpum* on 6-OHDA-induced damage on chicken brain slices was investigated in this study. 6-OHDA decreased cellular viability measured by MTT reduction assay, induced lipid peroxidation by HPLC, stimulated Glutathione-S-Transferase and Thioredoxin Reductase activity, and decreased Glutathione Peroxidase activity and the total content of thiols containing compounds. The methanolic fraction of *A. microcarpum* presented the better neuroprotective effects in 6-OHDA-induced damage in relation with hydroalcoholic and acetate fraction. The presence of AKT and ERK1/2 pharmacological inhibitors blocked the protective effect of methanolic fraction suggesting the involvement of survival pathways in the neuroprotection by the plant. The plant did not prevent 6-OHDA autoxidation or 6-OHDA-induced mitochondrial dysfunction. Thus, the neuroprotective effect of the methanolic fraction of *A. microcarpum* appears to be attributed in part to chelating properties of extract toward reactive species and is dependent on ERK1/2 and AKT phosphorylation. This study contributes to the understanding of biochemical mechanisms implied in neuroprotective effects of the vegetal species *A. microcarpum.*

## 1. Introduction

The Brazilian plant *Anacardium microcarpum*, popularly known as “cajui,” belongs to the Anacardiaceae family. It is found in the Northeast Region of Brazil and is used in traditional folk medicine for treatment of infectious diseases, inflammation, rheumatism, and tumor. Phytochemical constitution of *A. microcarpum* stem bark crude extract and fractions demonstrating the presence of different flavonoids such as gallic acid, caffeic acid, and quercetin [1]. Although a limited number of studies on this plant is available, our group demonstrated *in vitro* antioxidant potential and antibacterial effect of this plant [2].

Parkinson's disease (PD), which was described by James Parkinson in 1817, is the second most frequent neurodegenerative disease after Alzheimer's disease and is characterized by a progressive nigrostriatal neurodegeneration. This illness reaches all ethnic groups and socioeconomic classes and is present in approximately 1% of the world population over the 60s [3]. PD symptoms include resting tremor, stiffness, bradykinesia, and gait impairment [4]. The symptoms onset indicates an advanced stage of disease, with a substantial loss of dopaminergic cells in the substantia nigra and an 80% depletion of striatum dopamine [5]. It is known that genetic, environmental, and aging factors contribute to the progression of disease [6]. Moreover, biochemical factors such as oxidative stress, mitochondrial dysfunction, inflammation, and apoptotic cell death play important roles in the pathogenesis of PD [7, 8].

Substances able to damage selectively dopaminergic neurons are useful tools to study molecular mechanisms implied in neurodegeneration in PD and for screening of neuroprotective potential of chemicals. Among those substances are MPTP, paraquat, rotenone, and 6-hydroxydopamine [9].

6-hydroxydopamine (6-OHDA) is a toxic dopamine metabolite which is rapidly and nonenzymatically oxidized by molecular oxygen to form *p*-quinone and hydrogen peroxide [10] and is proposed as a putative neurotoxic factor contributing for PD pathogenesis. The induction of reactive oxygen species (ROS) formation is a major mechanism implied in neurotoxicity of 6-OHDA. Some characteristics of the brain tissue make it very susceptible to oxidative stress such as the elevated consumption of oxygen, high content of unsaturated fatty acids, and iron levels [11].

The most effective drug in the treatment of PD is L-DOPA; however, its frequent use is associated with neurotoxicity once L-DOPA gives rise to 6-OHDA via nonenzymatic reactions [12]. It is important to consider that the therapies available for PD delay the progression of degeneration and symptoms instead of providing an effective treatment for the disease. Thus, the search for alternative therapies such as natural antioxidants has grown greatly over the years; besides, there are evidence that plant extracts have beneficial potential, attenuating the progression of PD, through antioxidant compounds present in extracts [13–15]. The model of brain slices has provided an important contribution for detailing of brain circuits and neurochemical mechanisms and testing of the neuroprotective potential of compounds. The main factor why this model is considered appropriate for studying biochemical events in the brain is the maintenance of extracellular matrix, neuronal connectivity, and neuronal-glial interactions [16].

This study is aimed at evaluating the neuroprotective potential and the mechanisms that mediate the neuroprotection of *A. microcarpum* hydroalcoholic extract (AMHE), methanolic (AMMF), and ethyl acetate (AMEAF) fractions against 6-hydroxydopamine- (6-OHDA-) induced damage on cortical slices.

## 2. Materials and Methods

### 2.1. Chemicals

 Dimetil sulfoxide (DMSO), Folin-Ciocalteu, 2,2′-azino-bis(3-ethylbenzothiazoline-6-sulfonic acid diammonium salt, sodium acetate, HEPES minimum 99.5% titration, albumin from bovine serum (BSA), reduced glutathione (GSH), oxidized glutathione, tetramethylethylenediamine (TEMED), 3-(4,5-dimethylthiazol-2-yl)-2,5-diphenyltetrazolium bromide (MTT), D-Manitol, K_2_HPO_4_, KH_2_PO_4_, Triton X-100, *β*-mercaptoethanol, anti-rabbit immunoglobulin (HRP peroxidase-linked antibody), and carbonyl cyanide 4-(trifluoromethoxy) phenylhydrate (FCCP) were obtained from Sigma-Aldrich (São Paulo, SP, Brazil). SDS, acrylamide, bis-acrylamide, and hybond nitrocellulose were obtained from GE Healthcare Life Division (Uppsala, Sweden). Anti-phospho-p38 (Thr180/Tyr182) and total form, anti phospho-AKT (Thr308) and total form, anti-phospho PTEN, anti-phospho and total JNK1/2 (Thr183/Tyr185), anti-phospho ERK1/2 (Thr202/Tyr204) and anti-total-ERK1/2, and *β*-actin antibodies were purchased from Cell Signaling Technology (Danvers, MA). Poly (ADP)-ribose polymerase (PARP) antibody was obtained from Santa Cruz Biotechnology (Santa Cruz, CA). Kit Caspase-Glo 3/7 was obtained from Promega (Madison, WI). All other reagents were commercial products of the highest purity grade available.

### 2.2. Animals

For this study, it used chicks of *Gallus gallus* species from both genders with age among 5–15 days. The animals were maintained in the animal facility at controlled conditions of light and temperature with food and water ad libitum. All procedures were performed in accordance with the approval, under protocol no. 011/2012, of the CEUA/UNIPAMPA (Animal Ethics Committee from Universidade Federal do Pampa).

### 2.3. Plant Collection and Extractions

The stem barks of *A. microcarpum* were collected from Barreiro Grande, Crato-Ceará (7°22_S; 39°28_W; 892 m sea level), Brazil, in November 2011. The plant material was identified by Dr. Maria Arlene Pessoa da Silva of the herbarium Caririense Dárdano de Andrade-Lima (HCDAL) of the Regional University of Cariri (URCA), and a voucher specimen was deposited (n° 6702). The fresh barks of *A. microcarpum* were macerated with 99.9% of ethanol and water (1 : 1, v/v) for 3 days. The suspension was filtered, and the solvent evaporated and lyophilized under reduced pressure to obtain 490 g of hydroalcoholic extract. One hundred and fifty grams (150 g) of this was partitioned with ethyl acetate and methanol to obtain 12.5 g of ethyl acetate fraction and 105.23 g of the methanolic fraction. All fractions were stored in the freezer and resuspended in water prior to experiments.

### 2.4. Identification and Quantification of Phenolic Compounds and Flavonoids of *Anacardium microcarpum* by HPLC-DAD

The chemical composition of the *A. microcarpum* hydroalcoholic extract (AMHE), *A. microcarpum* methanolic fraction (AMMF), and *A. microcarpum* ethyl acetate fraction (AMEAF) was previously determined by our group [1] as shown in Table 1. The complete study can be found in literature where differences were verified between hydroalcoholic extract, methanolic, and ethyl acetate fractions, respectively.

### 2.5. Tissue Slice Preparation and Treatment

Animals were euthanized by decapitation under anesthesia. The brain was dissected and placed in cutting solution oxygenated at 4°C (110 mM sucrose, 60 mM NaCl, 3 mM KCl, 0.5 mM CaCl_2_, 7 mM MgSO_4_, 5 mM glucose, and 25 mM HEPES pH 7.4). The cortical region was separated, and 400 *μ*m thickness slices were prepared in a McIlwain tissue slicer [17]. The diameter of slices was standardized using a 3 mm punch. Briefly, slices were transferred to 96-well plates containing HEPES-saline buffer (124 mM NaCl, 4 mM KCl, 1.2 mM MgSO_4_, 12 mM glucose, 1 mM CaCl_2_, and 25 mM HEPES pH 7.4) previously oxygenated during 30 minutes (200 *μ*L/slice). After 30 min of preincubation, the buffer was removed, and fresh buffer was added. Tissue slices were subsequently incubated for 120 minutes at 37°C in the presence/absence of 6-OHDA 500 *μ*M and/or hydroalcoholic extract (AMHE), methanolic (AMME), and ethyl acetate *A. microcarpum* fractions (AMEA) (concentrations among 0.1–1 mg/mL). All dissolved in the culture medium.

### 2.6. Cell Viability

Cell viability was determined by the reduction of 3-(4,5-dimethylthiazol-2-yl)-2,5-diphenyltetrazolium bromide (MTT) (0.05% HEPES-saline). After 120 minutes of treatment, slices were incubated for 30 minutes at 37°C in the presence of MTT [17]. Subsequently, MTT was removed, and samples were incubated in DMSO for 30 min (37°C). The absorbance resulted from formazan product diluted in DMSO was read at 540 nm in an EnsPire® multimode plate reader (PerkinElmer, USA).

### 2.7. Spectrophotometric Studies of 6-OHDA Autoxidation

The autoxidation of 6-OHDA was followed by monitoring the formation of *p*-quinone at 490 nm [10]. A Cary 60-UV-Visible Spectrophotometer by Agilent Technologies was used for the assay. The cuvette holder was thermostatically maintained at 37°C. For each assay, 1 mL of phosphate buffer (pH 7.4) was incubated in a quartz cuvette for 10 min to reach the set temperature. Then, the autoxidation was initiated with the addition of 5 *μ*L of a stock solution of 6-OHDA (100 mM) at a final concentration of 0.5 mM. The monitoring of the corresponding kinetics was immediately initiated and maintained for subsequent 3 min. To verify if AMHE, AMMF, and AMEAF could prevent autoxidation of the compound, different concentrations of the plant extract or fractions (1 *μ*g/mL, 10 *μ*g/mL, and 100 *μ*g/mL) were added in the presence or absence of 6-OHDA. GSH 10 mM was used as positive control.

### 2.8. Lipid Peroxidation

The final product from lipid peroxidation was determined with thiobarbituric acid as the reactive substance (TBARS) with some modifications [18]. Tissue slices were incubated for 120 minutes at 37°C in different extract concentrations (0.1–1 mg/mL) in the presence/absence of 6-OHDA (500 *μ*M). For the next step, five slices per treatment group were homogenized in 150 *μ*L of HEPES 20 mM buffer. Further, all content was incubated during 60 minutes at 95°C into acetic acid/HCl 0.45 M buffer, thiobarbituric acid 0.8% (TBA), SDS 8.1% to promote the coloring, and absorbance was measured at 532 nm.

### 2.9. Enzyme Assays

Glutathione S-transferase activity (GST) was assayed using 1-chloro 2,4-dinitrobenzene (CDNB) as substrate [19]. The assay is based on the formation of the conjugated complex of CDNB and GSH. The reaction was conducted in a mix consisting of 0.1 M phosphate buffer pH 7.0, 1 mM EDTA, 1 mM GSH, and 2.5 mM CDNB. Glutathione peroxidase activity (GPx) was measured and defined as the rate of NADPH oxidation by the coupled reaction with glutathione reductase [20]. One unit of GPx will consume 1.0 *μ*mol of NADP^+^ from NADPH per minute (*ε* = 6.22 M^−1^ cm^−1^). Thioredoxin reductase (Trx-R) activity consists in measuring the rate of reduction of DTNB by NADPH [21]. One unit of enzyme activity was considered the amount of enzyme that catalyzes the formation of 1.0 *μ*mol of DTNB per minute at 25°C, pH 7.0 (*ε* = 13.60 M^−1^ cm^−1^). All spectrophotometric assays were performed at 340 nm in an Agilent Cary 60 UV/VIS spectrophotometer with an 18 cell holder accessory coupled to a Peltier Water System temperature controller (Santa Clara, CA).

### 2.10. Determination of Reduced (GSH) and Oxidized Glutathione (GSSG)

For the measurement of GSH and GSSG levels, brain homogenate was treated with 0.5 mL of 13% trichloroacetic acid and centrifuged at 100.000 g for 30 min at 4°C. Aliquots (10 *μ*L) of supernatant were mixed with 100 mM NaH_2_PO_4_ buffer, pH 8.0, containing 5 mM EDTA. *O*-phthalaldehyde (OPT) (1 mg/mL) was added, and fluorescence was measured 15 min later using the 350/420 nm excitation/emission wavelength pair in Perkin Elmer inspire [22]. For measurement of GSSG levels, brain supernatant was incubated at room temperature with N-ethylmaleimide (NEM) (0.04 M) for 30 min at room temperature, and after that, were added NaOH (0.1 N) buffer, following of added OPT and incubated for 15 min in the dark, using the procedure outlined above for GSH assay. Results were presented as the GSH/GSSG ratio.

### 2.11. High-Resolution Respirometry (HRR) *In Vitro*

For respirometry determination, chick brain (400 mg) was weighed and transferred to 1 mL of cold homogenization buffer containing 5 mM Tris-HCl, 250 mM sucrose, and 2 mM EGTA (pH 7.4), and brain homogenate was used to the HRR. Oxygraph-2k (O2k, OROBOROS Instruments, Innsbruck, Austria) was employed for all respiration measurements. Experiments were performed in 2 mL of MiR05 buffer (110 mM sucrose, 60 mM K-lactobionate, 0.5 mM EGTA, 3 mM MgCl_2_, 20 mM taurine, 10 mM KH_2_PO_4_, 20 mM HEPES pH 7.4, and 0.1% BSA) [23]. All experiments were performed at 37°C using DatLab 4.0 software (Oroboros Inc., Austria), with continuous stirring at 750 RPM, and all experiments started by registering the endogenous substrate supported respiration, following protocols established in the literature [24].

All experiments of mitochondrial bioenergetics analysis in brain homogenate were performed following [25] with minor modifications at the O2k-chamber. All concentrations of compounds (control group without treatment, 100 *μ*g/mL of a methanolic fraction, 6-OHDA 500 *μ*M in the absence or presence of methanolic fraction) were added at the O2k-chamber after signal stabilization of the basal respiration supported by endogenous substrates. Four individual preparations of the brain homogenate were performed per group.

### 2.12. Mitochondrial Respiration Assays

Titration protocols of multiple substrates and inhibitors were used to assess mitochondrial function in terms of different respiration states. The routine of electron transport system activities in brain homogenate was carried out according to literature [26]. Malate, glutamate, and succinate were used as oxidizable substrates in all experiments. Complex I- (CI-) mediated Leak (LEAK) respiration was determined using 2 mM malate and 10 mM glutamate. CI-mediated OXPHOS (OXPHOS) was determined using ADP (2.5 mM). Respiratory control ratios (RCR = CI_OXPHOS_/CI_LEAK_) were used as a quality control of isolated mitochondria. The convergent electron flow during the maximal OXPHOS respiration (CI + CII_OXPHOS_) was determined with substrates of CI and CII (10 mM Succinate). CI + CII-mediated ETS (electron transfer system) (CI + CII_ETS_) was determined using Carbonyl cyanide-*4*-(trifluoromethoxy) phenylhydrazone (FCCP) (optimum concentration reached between 0.5 and 1.5 *μ*M). CII-mediated ETS respiration (CII_ETS_) was determined with 0.5 *μ*M rotenone. Addition of 2.5 *μ*M antimycin A inhibited complex III, resulting in nonmitochondrial respiration (ROX) with small contributions from electron leak in the uncoupled state.

### 2.13. Western Blotting Analysis

Analysis of protein phosphorylation in cortical slices was performed using western blotting with slight modification [27]. Four slices were homogenized in 100 *μ*L of 4% SDS stop solution (4% SDS, 50 mM Tris, 100 mM EDTA, pH 6.8), and 10 *μ*L of sample was taken out for protein analysis. In the remaining sample was added 25% Glycerol sample and 8% *β*-mercaptoethanol. The proteins were separated by SDS–PAGE using 10% gels and then electrotransferred to nitrocellulose membranes. The membranes were washed in Tris-buffered saline with Tween-20 (100 mM Tris–HCl, pH 7.5, 0.9% NaCl, and 0.1% Tween-20) and incubated overnight at 4°C with primary antibodies anti-phospho p38, anti-total and phospho-ERK1/2, anti-total and phospho-JNK1/2, anti-phospho and total-AKT, anti-phospho PTEN. Subsequently, membranes were washed in Tris-buffered saline with Tween-20 and incubated for 1 hour at 25°C with horseradish peroxidase-linked anti-IgG secondary specific antibodies. The blottings were visualized on the IS4000MM Pro Bruker imaging system using ECL-detection reagent, and the band density was quantified using the Scion Image® software. The density of the bands was measured and expressed as a rate (%) of increase in relation to control (slices treated only with media).

### 2.14. PI3K/AKT and MEK Inhibitors

To determine the implication of signaling pathway involved in cell survival in the neuroprotective effect of *A. microcarpum* methanolic fraction, PI3K/AKT inhibitor LY294002 at final concentration of 20 *μ*M and MEK/ERK inhibitor PD98059 at final concentration of 50 *μ*M. PD98059 acts on inhibition of MEK1 in a reversible, allosteric, and noncompetitive manner with respect to ATP and ERK1/2 binding whereas LY294002 acts at an ATP-binding site of PI3K enzyme, thus selectively inhibiting the PI3K-Akt interaction. The inhibitors were added to the medium 30 min prior to the addition of 6-OHDA plus AMMF. After 2 hours of treatment, MTT assay was conducted to verify the slices viability as described above. Inhibitors were diluted in DMSO; the final concentration of DMSO in the wells was 0.5%.

### 2.15. Protein Quantification

The protein concentration in samples was estimated using BSA as standard [28, 29].

### 2.16. Statistical Analysis

All data were tested for normal distribution by Kolmogorov-Smirnov. Statistical analysis was performed using one-way analysis of variance (ANOVA) followed by Newman-Keuls post hoc analysis. Results were considered statistically significant when *p* < 0.05.

## 3. Results

### 3.1. Analysis of 6-Hydroxydopamine Autoxidation in the Presence of Extract and Fractions of *A. microcarpum*

**Figure 1:**
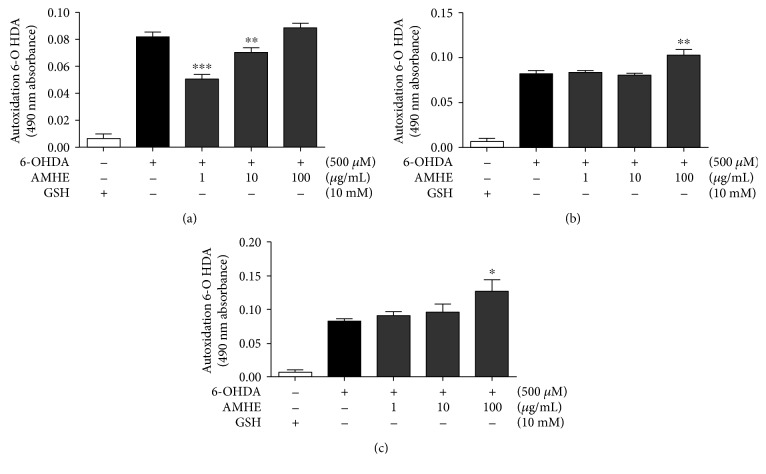
6-OHDA autoxidation in the presence of *A. microcarpum* and GSH. The autoxidation of 6-OHDA (500 *μ*M) was followed spectrophotometrically by monitoring the formation of *p*-quinone at 490 nm in the presence or absence of extract or fractions (a) 6-OHDA + AMHE, (b) 6-OHDA + AMMF, and (c) 6-OHDA + AMEAF. GSH 10 mM was used as positive control preventing 6-OHDA autoxidation. Data are expressed as percentage of the untreated control ± SE (*n* = 3). ^∗∗∗^*p* < 0.0001 as compared to GSH control. ##*p* < 0.001 and ###*p* < 0.0001 as compared to 6-OHDA group.

6-OHDA undergoes spontaneous autoxidation in the presence of oxygen under physiological conditions forming hydrogen peroxide (H_2_O_2_) and the corresponding *p*-quinone whose formation can be monitored spectrophotometrically at 490 nm [10]. The absorbance of 6-OHDA at the end of 3 min of incubation with phosphate buffer is visualized in the graph (Figures 1(a)–1(c)). The presence of fractions of *A. microcarpum* did not alter the 6-OHDA absorbance, whereas hydroalcoholic fraction decreased it partially. The antioxidant glutathione (GSH) was used as a positive control preventing 6-OHDA autoxidation due to the ability of sulfhydryl compounds to remove the H_2_O_2_ formed during the autoxidation reaction of 6-OHDA.

### 3.2. Evaluation of Toxicity and Neuroprotective Potential of *A. microcarpum* Hydroalcoholic Extract and Fractions

**Figure 2:**
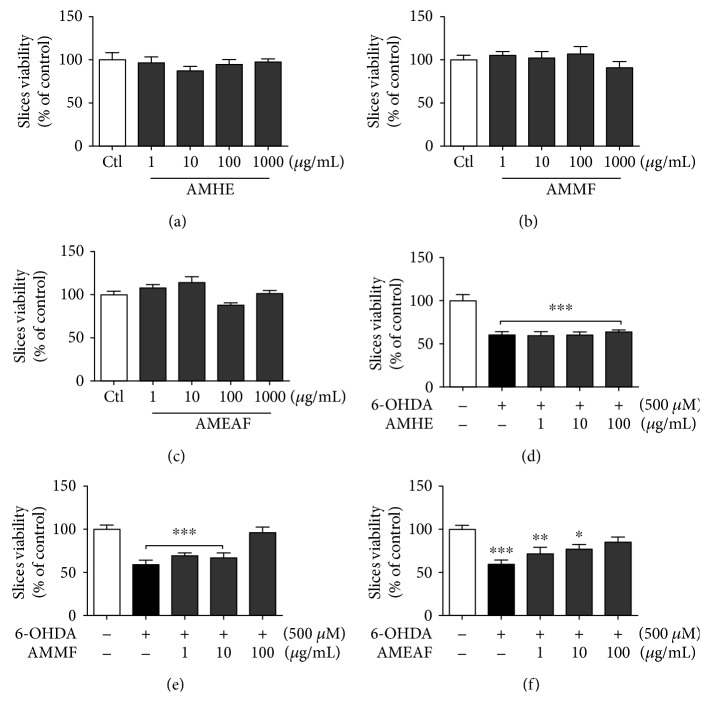
Effects of *A. microcarpum* and 6-OHDA on the viability of cortical slices. Cortical slices were incubated for 2 h in different concentrations (1–1000 *μ*g/mL) of (a) AMHE, (b) AMMF, and (c) AMEAF and in the presence or absence of 6-OHDA (500 *μ*M) during 2 h, (d) AMHE, (e) AMMF, and (f) AMEAF. Cell viability was measured by MTT test. Data are expressed as percentage of the untreated control ± SEM (*n* = 3). ^∗^*p* < 0.05, ^∗∗^*p* < 0.001, and ^∗∗∗^*p* < 0.0001 different from control group.

In order to investigate a possible neurotoxic effect of *A. microcarpum*, cortical slices were incubated for 2 hours with different concentrations of hydroalcoholic extract and fractions: 0, 1 *μ*g/mL, 10 *μ*g/mL, 100 *μ*g/mL, and 1000 *μ*g/mL. At the end of incubation period, cell viability assay was performed by MTT test. Our data showed that *A. microcarpum* per se was unable to affect the viability of slices (Figures 2(a)–2(c)).

To investigate the neuroprotective potential of *A. microcarpum*, slices were incubated with neurotoxin 6-OHDA 500 *μ*M for 2 hours in the presence or absence of different extracts or fraction concentration (1–100 *μ*g/mL). 6-OHDA concentration was defined in a dose-response curve, and the concentration able to decrease in approximately 30% the cell viability was chosen for further studies. The hydroalcoholic extract was unable to protect against damage caused by 6-OHDA (Figure 2(d)); however, AMMF and AMEAF reverted the drop in cell viability promoted by 6-OHDA at a concentration of 100 *μ*g/mL (*p* < 0.0001) (Figures 2(e) and 2(f), respectively).

### 3.3. Lipid Peroxidation in Response to the Treatment with Methanolic and Acetate Fractions of *A. microcarpum* and 6-OHDA

**Figure 3:**
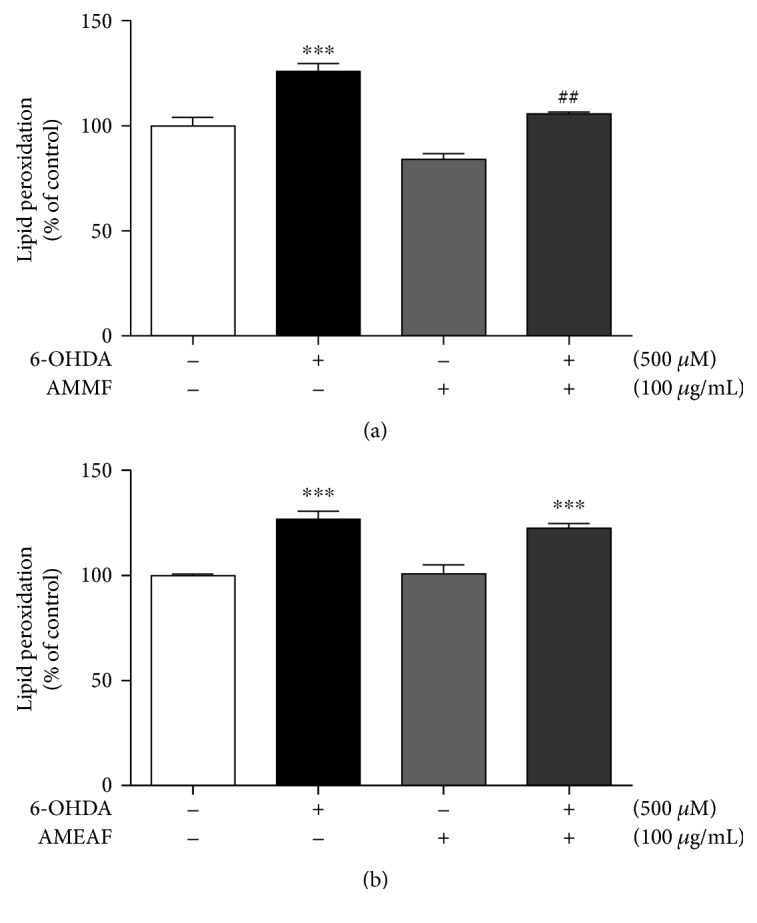
Effects of *A. microcarpum* on 6-OHDA (500 *μ*M) induced lipid peroxidation (LPO) in cortical slices. Cortical slices were incubated with (a) AMMF and (b) AMEAF in the presence/absence of 6-OHDA for two hours, and lipid peroxidation was evaluated by formation of TBARS at 532 nm. Data are expressed as percentage of the untreated control ± SEM (*n* = 3). ^∗∗∗^*p* < 0.0001 as compared to control. ##*p* < 0.001 as compared to 6-OHDA group.

Oxidative stress is implied in dopaminergic cell death induced by 6-OHDA [11]. AMMF and AMEAF but not AMHE presented neuroprotective potential in the MTT assay; thus, it was investigated a possible antioxidant potential of these fraction on slices exposed to 6-OHDA by its ability to prevent lipid peroxidation. 6-OHDA induced lipid peroxidation in 25% (*p* < 0.0001) when compared to control group. Only AMMF prevented this effect (Figures 3(a) and 3(b)); thus, further studies were conducted in the presence of methanolic fraction.

### 3.4. Analysis of ERK, AKT, PTEN Phosphorylation, and PARP Cleavage in Response to the Treatment with 6-OHDA and Methanolic Fraction of *A. microcarpum*

**Figure 4:**
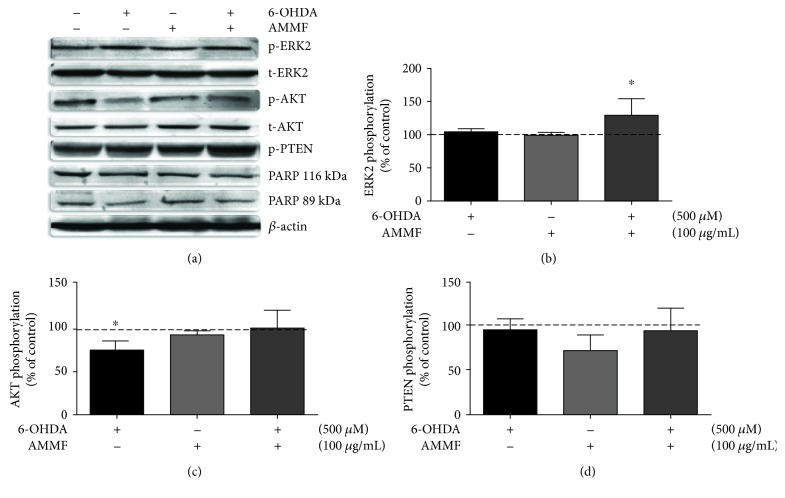
Modulation of ERK, AKT and PTEN phosphorylation, PARP cleavage in response to treatment with methanolic fraction of *A. microcarpum* and 6-OHDA in cortical slices. Proteins were separated by SDS–PAGE and transferred to nitrocellulose membrane. Total content and phosphorylation of proteins were detected by specific antibodies, and the reaction was developed by ECL. (a) Western blotting of phosphorylated and total forms of ERK2 and AKT phosphorylation and total forms and phospho-PTEN. (b) Quantitative analysis of ERK2 phosphorylation expressed as a ratio with its respective total form. (c) Quantitative analysis of AKT phosphorylation expressed as a ratio with its respective total form. (d) Quantitative analysis of PTEN phosphorylation expressed as a ratio with *β*-actin. The data are expressed as fold increase related to control group and represent mean ± SE of 4 independent experiments. Statistical analysis was performed by ANOVA, followed by the Newman-Keuls test. ^∗^*p* < 0.05, ^∗∗^*p* < 0.001, and ^∗∗∗^*p* < 0.0001 different from control group. #*p* < 0.05, ##*p* < 0.001, and ###*p* < 0.0001 when compared to 6-OHDA group.

In this study, the effect of 6-OHDA on phosphorylation of proteins p38, JNK1/2, ERK1/2, AKT, cleavage of PARP protein, and phosphatase PTEN was analyzed by western blotting technique. No alterations in phosphorylation and total levels of p38 and JNK1/2 were detected (data not shown). The phosphorylation of ERK was increased in 30% only in the presence of extract and 6-OHDA (Figure 4(b)); no alterations were observed in the other groups. AKT phosphorylation (Figure 4(c)) was inhibited in approximately 25% by 6-OHDA treatment and remained at control level when fraction was present. The cleavage of PARP protein in an 89 kDa fragment was evaluated as an indicator of apoptotic cell death. No alteration in PARP cleavage was visualized by treatments as observed in the blotting. PTEN phosphorylation was unchanged by the treatments (Figure 4(d)).

### 3.5. Involvement of ERK and AKT Signaling Pathways in the Neuroprotective Mechanisms of Fraction

**Figure 5:**
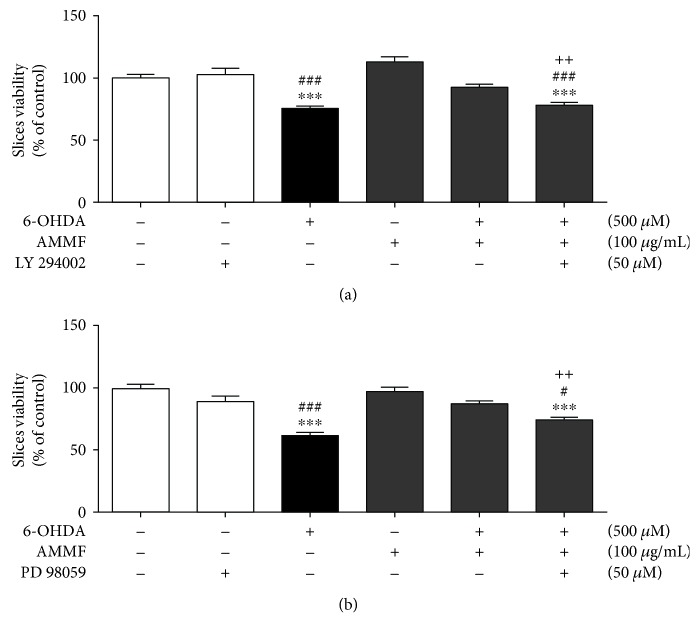
Effects of *A. microcarpum* and 6-OHDA on the viability of cortical slices in the presence of LY (294002) inhibitors and PD (98059). Slices were incubated with AMMF in the presence or absence of 6-OHDA (500 *μ*M) and inhibitors for 2 h. The inhibitors were added 30 min prior to the addition of fraction and 6-OHDA and remained during all period of the treatment. Data are expressed as percentage of the untreated control ± SE (*n* = 3). ^∗∗∗^*p* < 0.0001 as compared to control. ###*p* < 0.0001 as compared to only inhibitor treated group; ++*p* < 0.001 as compared to cotreated group 6-OHDA + AMMF.

The participation of ERK1/2 and AKT in the protective potential of the methanolic fraction was investigated. Slices were incubated with synthetic inhibitors of ERK1/2 phosphorylation (PD98059) and AKT phosphorylation (LY294002) for 30 min prior to the addition of 6-OHDA or methanolic fraction. As shown in Figure 5, the inhibition of ERK and AKT blocked the protective effects of extract.

### 3.6. Activity of Antioxidant Enzymes and Redox State of Cells in Response to the Treatment with the Methanolic Fraction of *A. microcarpum* and 6-OHDA

As shown in Table 2, 6-OHDA caused a 1.68-fold increase in GST activity, and this effect was not observed in the presence of methanolic fraction and 6-OHDA. 6-OHDA induced TRx-R activity in 1.9-fold; this effect was not observed in the presence of methanolic fraction. On the other hand, GPx was inhibited in 1.71-fold by 6-OHDA when comparing to control, and this effect was not observed in the presence of methanolic fraction. The total glutathione content and oxidized glutathione were decreased by 34% and 39%, respectively, by 6-OHDA treatment, and the ratio GSH/GSSG was increased by 6-OHDA. This effect was not observed when 6-OHDA was present. The plant per se increased levels of reduced GSH (Table 3).

### 3.7. Mitochondrial Respiration in Response to 6-OHDA and *A. microcarpum* Methanolic Fraction

**Figure 6:**
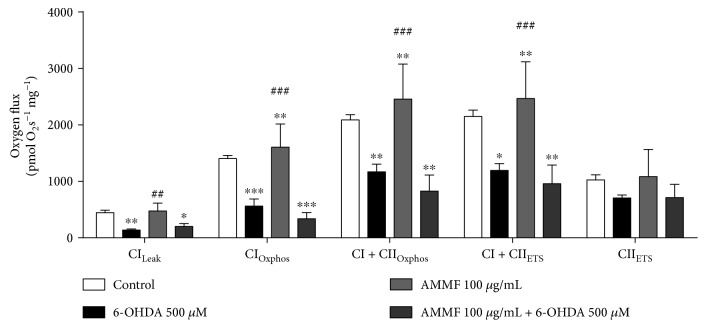
O_2_ flux measured in the mitochondria of cortex homogenate exposed to 6-OHDA and/or *A. microcarpum.* Mitochondrial function is presented with the abbreviation(s) of the complex(es) involved followed by the state of respiration measured in the presence of glutamate + malate (CI_LEAK_), +ADP (CI_OXPHOS_), +succinate (CII_OXPHOS_), +FCCP (CI + II_ETS_), +rotenone (CII_ETS_). Antimycin A was used to correct for residual O_2_ consumption. Results are means ± SEM for 4 different preparations. ^∗^*p* < 0.05, ^∗∗^*p* < 0.001, and ^∗∗∗^*p* < 0.0001 different from control group. ##*p* < 0.001 and ###*p* < 0.0001 when compared to 6-OHDA group.

The mitochondrial respiration in response to 6-OHDA and methanolic fraction was measured in brain homogenate by cellular oxygen consumption. Basal respiration was unchanged in the brain by treatments (data not shown). After glutamate and malate substrate (CI_Leak_) addition, a significant decrease (*p* < 0.05) on CI activity was induced by 6-OHDA. This drop in CI activity persisted when methanolic fraction was added (*p* < 0.05). AMMF per se did not change the activity of CI. In order to see CI_OXPHOS_, it was added succinate and ADP (CI_OXPHOS_); this parameter was also inhibited by 6-OHDA, and the fraction was unable to avoid it. The convergent electron flow during the maximal oxidative phosphorylation (CI + CII_OXPHOS_) was also significantly decreased by 6-OHDA, and the fraction did not avoid this effect. Maximal mitochondrial respiration (CI + CII_ETS_) was determined with the addition of the uncoupler FCCP. This parameter was inhibited by 6-OHDA and not avoided by the fraction (*p* < 0.001). CII_ETS_ activity was analyzed after inhibition of CI by rotenone, and no significant changes occurred by treatments. AMMF per se but not in the presence of 6-OHDA induced CI_LEAK_, CI_OXPHOS_, CI + CII_OXPHOS_, and CI + II_ETS_ (Figure 6).

## 4. Discussion

Oxidative stress, mitochondrial dysfunction, genetic, and environmental factors are mechanisms associated with neuronal damage observed in PD [30–32]. A number of studies have proposed antioxidant therapies to attenuate PD symptoms [33, 34]. In this study, methanolic and acetate fraction but not hydroalcoholic extract protected against neurotoxicity induced by 6-OHDA in brain slices, but methanolic fraction was more effective in protecting against induction in lipid peroxidation by 6-OHDA.

The cytotoxic effect of 6-OHDA is attributed to the formation of reactive species such as superoxide radical, *para*-quinone, and hydrogen peroxide from enzymatic and autoxidation reactions [35]. It this study, plant extract or fractions did not block effectively the autoxidation of 6-OHDA *in vitro*. With the base in this finding, it can be inferred that the mechanism implied in the protective effect of the plant is the neutralization of reactive species secondary to the autoxidation reaction and not a direct interaction with 6-OHDA molecule. In previous studies, 6-OHDA induced mitochondrial dysfunction by inhibition of I and IV complexes, disrupting the mitochondrial function, and producing superoxide anion, which in turn may form hydroxyl radicals which reacts with nitric oxide generating peroxynitrite [36–39]. In the present study, the 6-OHDA inhibited in 55% complex I activity in basal respiration around 75% the ATP production CI-dependent and compromise the mitochondrial electron transport system in 50%. This is in accordance with a similar work that showed a 20% of inhibition of complex I at a concentration of 100 *μ*M, approximately [40]. This phenomenon was not blocked by *A. microcarpum.* Considering the inability of the plant to hamper the mitochondrial damage caused by 6-OHDA, it could be supposed that the neuroprotective effect by the plant is due to the neutralization of reactive species resulted from mitochondrial dysfunction.

Reactive oxygen species production, detoxification, and signaling pathways have been considered interesting targets for intervention in neurodegenerative diseases [41, 42]. Endogenous enzymatic and nonenzymatic antioxidants, such as GSH, glutathione S-transferase, glutathione peroxidase, and thioredoxin reductase (TRx-R), delay or prevent oxidative damage to proteins, lipids, and DNA [43, 44]. Glutathione peroxidase (GPx) is an intracellular antioxidant that reduces hydrogen peroxide to water at expenses of GSH and limits its harmful effect. In this study, 6-OHDA caused a substantial inhibition in GPx activity that was not observed in presence of methanolic fraction. Similar data were demonstrated in neuroblastoma cells treated with 6-OHDA [45]. In PD patients, the degree of symptom severity correlates with intracellular GSH loss in substantia nigra [46]. In our study, GSH content was not altered, and GSSG was decreased by 6-OHDA; this effect may be related to GPx inhibition and consequently a lower oxidation of GSH by this system. On the other hand, the TRx-R activity was stimulated by 6-OHDA, and the same data were observed in neuroblastoma cell line SH-SY5Y [47]. Our results suggest the participation of peroxiredoxins catalyzing peroxide reduction as a compensatory mechanism to replace the inhibited activity of GPx. Herein, 6-OHDA increased the activity of GST, which is implied in neuronal detoxification of quinones resulted from catecholamine oxidation and free radicals [48], and this effect was not observed when plant was present. All these effects were prevented by the methanolic fraction of plant, showing a protective mechanism against oxidative stress induced by the 6-OHDA. Due to methodological issues, we were not able to detect quantifiable levels of the enzymes Superoxide Dismutase and Catalase, but their analysis will be considered in further studies.

Extracellular signal-regulated kinases (ERKs) have been implicated in the cellular response to reactive oxygen species [49–52]. Growth factors and other extracellular stimuli activate the kinase MEK1/2 by Ras/Raf pathway; MEK1/2 then phosphorylates and activates ERK1/2 [53]. ERK1/2 activates transcription factors such as cAMP response element–binding protein (CREB) and Elk, thereby increasing transcription of neurotrophic factors and prosurvival genes such as Bcl-2 [54]. In this study, the use of MEK1/2 inhibitor weakened the protective potential of methanolic fraction against the 6-OHDA, suggesting that the antioxidant potential of fraction per se is not enough to protect the brains slices, but the activation of prosurvival factors plays an important role in this effect. Experiments of western blotting showed that 6-OHDA did not alter ERK phosphorylation in brain slices after two hours of incubation with 6-OHDA. Previous studies reported an ERK1/2 phosphorylation peak after 10–15 min of exposure of dopaminergic cells to 6-OHDA; the phosphorylation of prosurvival protein CREB followed this temporal profile as well [54]. Authors showed that the inhibition of early phosphorylation of ERK1/2 abolished CREB activation and increased 6-OHDA toxicity. Thus, the possibility of an early activation of ERK1/2 in slices submitted to the treatment and the contribution of this activation for a self-protective response of cells that was prevented with use of inhibitors could not be discarded.

AKT is a serine/threonine kinase and its signaling pathway plays an important role in fundamental cellular functions, such as cell proliferation and survival, by phosphorylating a variety of enzymes, including proapoptotic regulators, antioxidant proteins, and transcription factors [55]. It is reported that AKT phosphorylation is reduced in the striatum of patients with PD, suggesting that its inactivation has an important role in PD [56]; being so, it is a substantial therapeutic target for treating neurodegenerative diseases, beyond other pathologies [57]. Herein, 6-OHDA inhibited phosphorylation level of AKT, and the plant prevented this effect. There was no alteration in phosphorylation level of PTEN (Phosphatase and tensin homolog deleted on chromosome ten), a negative regulator of AKT. Similar results were reported in SH-SY5Y and dopaminergic cell lines [58, 59]. In this study, AKT phosphorylation seems to display a role in neuroprotective effect of fraction against 6-OHDA, once the use of PI3K/AKT inhibitor blocked the protective effect of fraction. This result proposes that survival signaling pathways ERK and AKT contribute for neuroprotection by methanolic fraction of *A. microcarpum.*

Recent studies have suggested that several signal transduction pathways, including phosphatidylinositol 3 kinase (PI3K) pathways and MAPKs, are involved in releasing transcription factor Nrf2 from the complex Keap1-Nrf2 promoting Nfr2 translocation to the nucleus [60]. Nrf2 promotes transcriptional activation of a variety of antioxidant genes [61]. AMMF-mediated cytoprotection against 6-OHDA was abolished by ERK and AKT pathway inhibitors; these data support a possible involvement of Nrf2 activation leading to expression of downstream antioxidant genes through the modulation of AKT and ERK1/2 pathways by the fraction. According to another study, inhibition of AKT and ERK has also abolished the neuroprotective effect of a triterpenoid isolated from plant [62].

## 5. Conclusion

The present work shows for the first time the potential of the Brazilian plant *A. microcarpum* in protecting against 6-OHDA-induced damage in brain slices. Inhibition of mitochondrial complexes by 6-OHDA was not avoided by the extract. ERK and AKT phosphorylation display a role in the neuroprotective effect by the plant which was decreased by the presence of pharmacological inhibitors of those pathways.

## Figures and Tables

**Table 1:** Phytochemical characterization of extract and fractions of *Anacardium microcarpum.* (Adapted from Barbosa-Filho et al., 2014.)

Compounds	AMHE (mg/g)	AMMF (mg/g)	AMEAF (mg/g)
Gallic acid	14.53 ± 0.02	7.13 ± 0.01	21.32 ± 0.04
Chlorogenic acid	5.83 ± 0.03	—	10.57 ± 0.03
Caffeic acid	19.36 ± 0.02	13.57 ± 0.05	27.19 ± 0.03
Ellagic acid	15.12 ± 0.01	13.19 ± 0.01	25.61 ± 0.05
Catechin	3.79 ± 0.01	3.05 ± 0.04	6.24 ± 0.02
Epicatechin	4.53 ± 0.01	3.11 ± 0.01	9.35 ± 0.01
Rutin	3.81 ± 0.03	9.86 ± 0.03	7.03 ± 0.01
Isoquercitrin	14.25 ± 0.01	15.79 ± 0.03	25.98 ± 0.02
Quercetrin	7.29 ± 0.02	13.20 ± 0.02	20.64 ± 0.02
Quercetin	28.03 ± 0.04	18.16 ± 0.01	27.02 ± 0.01
Kaempferol	3.54 ± 0.01	9.93 ± 0.02	11.25 ± 0.02
Kaempferol glycoside	9.06 ± 0.03	3.15 ± 0.04	3.47 ± 0.01

**Table 2:** Activity of antioxidant enzymes in cortical slices submitted to treatment with the neurotoxin 6-OHDA and *A. microcarpum* methanolic fraction.

	GST (mg/mU protein)	GPx (mg/mU protein)	TrxR (mg/mU protein)
Control	241.2 ± 8.49	41.37 ± 5.01	2.375 ± 0.342
6-OHDA 500 *μ*M	406.4 ± 75.89^∗^	23.82 ± 2.07^∗^	4.675 ± 0.608^∗^
AMMF 100 *μ*g/mL	186.2 ± 42.13^#^	51.01 ± 4.04	2.459 ± 0.137^##^
AMMF 100 *μ*g/mL + 6-OHDA 500 *μ*M	251.5 ± 36.48^#^	46.29 ± 3.20^##^	3.164 ± 0.496^#^

Data are expressed as percentage of the untreated control ± SEM. ^∗^*p* < 0.05 in relation to control group, #*p* < 0.05 in relation to control group, ##*p* < 0.001 in relation to 6-OHDA group.

**Table 3:** Effect of treatment with 6-OHDA and *A. microcarpum* on GSH and GSSG levels and ratio GSH/GSSG.

	GSH (% of control)	GSSG (% of control)	Total glutathione (% of control)	GSH/GSSG (% of control)
Control	88.05 ± 6.73	90.60 ± 5.48	100.0 ± 10.66	100.0 ± 2.51
6-OHDA 500 *μ*M	87.49 ± 5.41	61.61 ± 12.87^∗^	66.56 ± 11.34^∗^	168.1 ± 19.64^∗∗^
AMMF 100 *μ*g/mL	136.7 ± 6.54^∗∗^	117.2 ± 10.04^##^	129.7 ± 4.18^##^	120.7 ± 9.21^##^
AMMF 100 *μ*g/mL + 6-OHDA 500 *μ*M	111.4 ± 13.28	111.9 ± 3.17^##^	111.8 ± 4.96^#^	99.61 ± 8.99^##^

Data are expressed as percentage of the untreated control ± SEM. ^∗^*p* < 0.05; ^∗∗^*p* < 0.001 in relation to control group. ##*p* < 0.001 when compared to 6-OHDA group.

## Data Availability

The data used to support the findings of this study are available from the corresponding author upon request.
